# Exploring the feasibility, acceptability and value of volunteer peer mentors in supporting self-management of osteoarthritis: a qualitative evaluation

**DOI:** 10.1080/09638288.2021.1964625

**Published:** 2021-09-09

**Authors:** Elizabeth C. Lavender, Esther Dusabe-Richards, Anna M. Anderson, Deborah Antcliff, Linda McGowan, Philip G. Conaghan, Sarah R. Kingsbury, Gretl A. McHugh

**Affiliations:** a School of Healthcare, University of Leeds, Leeds, UK; b Leeds Institute of Rheumatic & Musculoskeletal Medicine, University of Leeds, and NIHR Leeds Biomedical Research Centre, Leeds, UK; c Physiotherapy Department, Bury & Rochdale Care Organisation, Northern Care Alliance NHS Group, Salford, UK

**Keywords:** Peer mentors, osteoarthritis, self-management, older volunteers, qualitative study

## Abstract

**Background:**

Hip and knee osteoarthritis (OA) affect a large and growing proportion of the population. Treatment options are typically conservative making self-management a priority. Using trained peers to support individuals with OA has potential to improve self-management.

**Purpose:**

To explore the process of engaging and training volunteers to become peer mentors; and to qualitatively evaluate the feasibility, acceptability and value of being a peer mentor to support others’ self-management of OA.

**Materials and methods:**

A qualitative evaluation of a peer mentorship support intervention reporting the processes of recruitment and training; and semi-structured interviews conducted with nine active peer mentors. Transcribed interviews were coded and analysed using framework analysis.

**Results:**

It was possible to recruit, train and retain volunteers with OA to become peer mentors. The peer mentors benefitted from their training and felt equipped to deliver the intervention. They enjoyed social elements of the mentorship intervention and gained satisfaction through delivering valued support to mentees. Peer mentors perceived the mentorship intervention to have a positive impact on self-management of OA for mentees.

**Conclusion:**

Training volunteers with OA to become peer mentors was feasible and acceptable. Peer mentors perceived their support benefitted others with OA. They positively rated their experience of providing mentorship support.

**IMPLICATIONS FOR REHABILITATION**
- This study demonstrates that it is possible to recruit, train and engage older volunteers to become peer mentors for people with osteoarthritis.
- Training should highlight the significance of employing key self-management techniques such as goal-setting.
- Peer mentors acknowledged that they benefitted from training and delivering the mentorship intervention, and this impacted positively on their own osteoarthritis self-management.
- Careful consideration of matching mentors and mentees appears to enhance the success of mentorship support.
- Recognising the impact of mentorship support on mentees’ self-management is central to peer mentors’ sustained engagement with the intervention.

## Introduction

Osteoarthritis (OA) is a common long-term condition most prevalent in older people [1]. Around one in 10 adults in the UK have been diagnosed with OA by a GP, with knees and hips being most commonly affected sites [2]. The prevalence of OA is growing with longer life-expectancy, increasingly sedentary lifestyles and rising obesity levels [3]. Symptoms include pain, stiffness, and reduced mobility with a consequent risk of comorbidities [4,5]. Quality of life may be severely affected [6,7]. OA presents substantial individual health and socio-economic burdens [8–11], and indirect costs are largely underestimated [10,12]. Management of OA is difficult, and treatment options tend to be conservative [13]. A shift towards improving self-management of long-term conditions such as OA has been recognised by policy makers as the preferred way forward [14–16].

Self-management of OA focuses on enabling individuals to take control of their symptoms through acquiring knowledge and skills to lessen the impact of everyday challenges [17]. Through mutually informed and shared understanding of the condition, patients can become active partners, collaborating and communicating more effectively with health professionals and others to achieve desired outcomes and access appropriate support. Central to the success of self-management of long-term conditions is maintenance of health behaviours which can be enhanced by effective goal-setting and peer support [18,19]. People who share a common health condition, often referred to as “peers,” provide a unique reciprocal resource [20]. Older volunteers have much to offer in terms of their life experience, understanding and willingness to help. Their involvement often improves patient experience, enhances engagement in hard-to-reach groups and bridges the gap between services and communities [21,22].

Volunteers themselves may benefit from offering support, through improvements in their knowledge, confidence, skills and health outcomes [23]. One study of volunteers delivering an arthritis self-management group reported increased self-efficacy and improvements in their own symptoms (pain, fatigue, mood) when observing positive changes in course participants [24]. They attributed these mutual benefits to social involvement and active participation in the course.

Peer mentorship is a self-management model focusing on one-to-one, person-centred support. Models of peer mentorship have been explored for long-term conditions such as cancer, diabetes and chronic low back pain (CLBP) [25–29]. Models which are individualised and responsive to target group needs, indicate improvements to quality of life, coping-efficacy, and sense of agency amongst mentees [20,30,31]. Studies show that peer mentorship can effectively improve glucose control in people with diabetes [29,32]; improve pain management in people with heart disease [33]; and can provide a cost-effective alternative to usual care [31,32,34]. Peer mentorship appears to be beneficial in self-management of long-term inflammatory conditions such as Systemic Lupus Erythematosus (SLE) and Early Inflammatory Arthritis (EIA) [9,30,35]. It is likely to be particularly appropriate for people with OA, as symptoms are variable, changeable and persistent. However, there are no previous studies investigating a peer mentorship intervention for people with OA.

Previous research suggests that there are key elements that enhance the success of self-management in peer mentorship interventions [23,36–38]. Walshe et al. describe recruitment and training of peer mentors with advanced cancer as “*critically important to the planned intervention”* [25]. Understanding the mechanisms of recruitment, the motivation of volunteers and potential attrition are essential foundations for subsequent training and intervention delivery. Evidence from other peer mentorship studies highlights the importance of refining recruitment inclusion criteria to reduce attrition, and of careful consideration of mentors’ preferences for matching [19,39,40]. Matching peer mentors with mentees, while an important component of personalised support, is *“an inexact process”* [36]. Although consideration of matching preferences are important for mentor acceptability, they may not be the critical criteria for intervention success [40]. The research literature suggests that matching criteria are salient to mentorship support [19,35,36], and lived experience appears to be more significant than traditionally used demographic characteristics. Sandhu et al. (2013) proposed that matching based on gender, personal, and social characteristics facilitates inter-personal connections [30].

Given the potential benefits of engaging volunteers in peer support, and the impetus for self-management as a policy mechanism for people with long-term conditions, we wanted to explore whether peer mentorship support has the potential to be effective for OA self-management in older people.

## Study design, aims and objectives

The overall aim of the study was to develop and trial a peer mentorship support intervention to improve self-management amongst older people with hip/knee osteoarthritis, and determine the feasibility of conducting a definitive randomised controlled trial (RCT). Using the Medical Research Council guidance on developing and evaluating complex interventions [41] as a theoretical structure we developed, piloted and evaluated the peer mentorship support intervention as a two-arm randomised feasibility trial, reported elsewhere [42], with a nested qualitative evaluation, reported here. Key objectives were to assess feasibility and acceptability of the intervention and trial procedures, including: mentor and mentee recruitment and retention; mentor training; intervention costs; completion rates; and potential impacts of the intervention on participants.

Specifically the two aims of the qualitative evaluation reported here were: (1) to explore and understand the process of engaging community-based volunteers with OA and training them to become peer mentors; and (2) to explore the feasibility, acceptability and value of the intervention from the peer mentors’ perspectives through a qualitative interview study.

Specific objectives were:

1. To explore whether it is feasible to recruit and train older volunteers to become peer mentors for people with OA within a research study.
2. To explore how and why participating in the intervention may be acceptable to peer mentors.
3. To explore peer mentors’ perceptions of the impact of their support on self-management of older people with OA.

The dual purposes of this paper are therefore to report on the process and experience of *becoming* a peer mentor through engagement and training; and mentors’ perspectives of *being* a peer mentor for people with OA, as captured through qualitative interviews.

## Materials and methods

The study was located in a large city in the north of England where 50% of the population are aged 50+ years. Ethical approval was granted by Greater Manchester South Research Ethics Committee (Reference:17/NW/0238).

### Becoming a peer mentor

#### The role

The role of volunteer peer mentor for this study was to deliver informational, practical and emotional support to mentees using educational resources and self-management techniques that were provided through training. Additionally, the role required mentors to share their experience of living with OA, particularly their own self-management strategies and outcomes. To be eligible for the role, volunteers needed to be aged 50+ and have hip and/or knee OA.

Mentors were required to complete 12 hours of training prior to delivering up to eight one-hour, face-to-face mentorship support sessions with a mentee. As sessions generally took place in the mentee’s home, mentors needed to be able to travel independently. They were asked to record session content and progression on summary sheets.

#### Engaging volunteers as peer mentors

We intended to recruit eight volunteers to provide mentorship support to 25 intervention participants (individuals with OA randomly allocated to receive peer mentor support). However, the previous literature identified potential difficulties in recruiting (such as suitability and capability to fulfil the role), and retaining peer mentors who themselves are self-managing long term conditions [25,29,39], we therefore revised our target recruitment to 10–12 volunteers. We anticipated that in addition to self-managing their OA, volunteers may have other physical or mental health comorbidities, discover incompatibility with the role, or limitations of time and availability, all of which could lead to attrition. We considered the age and lived experience of hip/knee OA to be important features of a “peer” for study participants.

Volunteers were recruited through posters displayed in hospital outpatient clinics and workplaces; social media posts; direct appeal to 35 community and church groups, via 10 General Practitioner and physiotherapy practices; and advertising through a volunteering website and a community magazine. Potential applicants were invited to contact the study team. The Volunteer Coordinator (VC) responsible for supporting mentors followed up all enquires both to explain the purpose of the study, peer mentor role, and training; and to assess the appropriateness of applicants. Those interested were asked to complete a short application form and supply two referees. On receipt of satisfactory character references, applicants were sent written confirmation and information about training. The study team used training days as an opportunity to further assess applicants’ suitability as peer mentors. Similarly, applicants used training days to decide whether they wished to proceed. All were assisted with their Disclosure and Barring Service (DBS) applications and issued with Letters of Access. Mentors signed a consent form adhering to confidentiality, data protection and lone working policies.

#### Training volunteers to become a peer mentor

Successful applicants were required to attend one of three, 2-day training events run between September 2018 and February 2019. The training programme was developed by the study team with input from health professionals and PPI members. The first training event was led by representatives of Arthritis Care Northern Ireland (now subsumed under Versus Arthritis) and ran on consecutive days. Incorporating feedback from initial training, subsequent training included a weekend gap between the two days. This break reduced the intensity of sessions and created an opportunity for volunteers to become familiar with study resources. All training was delivered jointly by members of the multi-disciplinary study team (researchers, a physiotherapist and nurse) and included input from a specialist Musculoskeletal (MSK) physiotherapist. Members of the Patient and Public Involvement (PPI) group supporting the study were also invited to participate.

The training programme incorporated a mixture of theoretical, interactive and group work sessions. Content covered OA-related topics such as muscle-strengthening, pain management and pacing; development of mentoring skills (active listening, goal-setting); using resources; and mentoring in practice (safeguarding, lone working and session recording). Volunteers were given an educational resource manual and handouts to use in support sessions with mentees. Additional information about peer mentor training and associated costs can be found in the feasibility trial paper [42].

#### Matching mentors and mentees

The original criteria for matching mentors with mentees were location, gender, age and OA site(s). Matching criteria were subsequently adapted due to mentor availability and preferences. Some criteria, such as life circumstances and personality characteristics, were prioritised over others, such as location, although travel convenience was considered.

Prior to being matched, mentors were asked for their preferred days/times for sessions. Several mentors were restricted by forthcoming holidays and medical procedures, one requested not to be matched with an opposite gender participant, and two had animal allergies. All mentors drove their own cars although some were reluctant to travel too far, or at peak times. These constraints were taken into account as far as practicable, although in response to time pressures and the number of participants waiting to be supported, some mentors became more flexible with their matching preferences.

The intervention was designed for mentors to support one person at a time. However, when this became a logistical challenge with limited time left to complete the feasibility trial, two mentors agreed to support two participants concurrently.

### The peer mentor intervention

Trained and safety-checked mentors went on to be matched with feasibility trial participants (people with hip and/or knee OA who had been randomised into the intervention group). Using their training, the study resource manual, and their lived experience of OA, mentors supported between one and four mentees in weekly 1-hour sessions for up to 8 weeks. Sessions took place in the mentee’s home or private workplace at a previously arranged time. Following our lone-working policy, mentors used a buddy system, checking in and out of sessions via text/phone with the Volunteer Coordinator or other staff member. This contact enabled mentors whereabouts to be tracked and provided opportunities for one-to-one support and guidance. Regular group support with other mentors was available throughout the intervention. After each session mentors recorded progress, goals and challenges. Following NIHR INVOLVE guidance, mentors were paid honorarium for each support session, and all travel expenses incurred during training and mentoring were reimbursed.

### Recruitment to the qualitative study

At the end of the intervention, all mentors who were actively engaged with the study were invited to participate in qualitative interviews to give their perceptions of the mentoring process. With their permission they were contacted by a researcher who was previously unknown to them. Mentors who agreed to be contacted were sent a Participant Information Sheet and covering letter via email or post. This information explained that the purpose of the interview was to gain mentors’ views on the intervention, and to explore their individual experiences of providing peer mentorship to people with hip/knee OA.

## Data collection

Semi-structured interviews took place, in private at the participants’ home or workplace, between June and November 2019. The familiar setting for the interviews enabled participants to feel at ease. Interviews were conducted face-to-face by an experienced independent qualitative researcher (EDR) to encourage openness to questions about training, study procedures and support. The interviews focused on the key elements of feasibility and acceptability, and mentors’ perceptions on the potential impact of intervention support on mentees’ self-management (Table 1).

**Table 1.** Peer mentor interview topics

Recruitment of volunteers:
Volunteering for the role of peer-mentor
Motivation to apply to the role
Application process, communication and improvements
Training to become a peer-mentor:
Training experience including fellow trainees
Training structure, content, delivery and improvements
Personal usefulness of training
Effectiveness of training for peer-mentorship
Peer mentoring intervention delivery:
Exploring matching process and matching experience
Experience of session delivery
Exploring self-management support for mentees
Perceived impact of self-management support for mentees
Peer mentoring intervention processes:
Feeling supported as a peer-mentor: study team
Feeling supported as a peer-mentor: fellow peer-mentors
Future volunteering as a peer-mentor

Prior to obtaining written consent, the researcher clarified confidentiality and data management procedures and sought permission to audio-record the interview. All recordings were uploaded to a secure file immediately following the interview and sent for transcription. Returned transcripts were anonymised prior to analysis.

## Data analysis

Interview transcripts were analysed following a Framework Analysis approach [43,44] to enhance transparency and objectivity. Familiarisation and systematic open coding of the interview data was undertaken separately by two experienced qualitative researchers (EL and EDR), one who had been closely involved with the mentorship intervention and one who was independent. This inductive process enabled identification of themes which formed the basis of the analytical framework used for charting. Themes arising from the interview topics were further categorised into components of the mentoring process, which aligned to feasibility; and mentoring in practice, which aligned to acceptability. Key themes were presented as preliminary findings at an early dissemination event with study stakeholders, providing opportunities for discussion, development and refinement.

Procedures to ensure rigour and trustworthiness were adhered to throughout data analysis. The rich interview data was subject to repeated reflexive interrogation by independent researchers, individual interpretations were checked for meaning and potential bias by members of the wider research team, and any discrepancies were discussed. Internal validity was further ensured by triangulation of mentor interview data with mentee interview data and accumulated knowledge from prolonged engagement with mentors.

## Results

### Engagement and training of peer mentors

Mentor recruitment took place between May 2018 and January 2019. Thirty-two people expressed interest in the role: 11 were ineligible and five were unavailable on training dates. Reasons for ineligibility included not having hip or knee OA and inability to travel independently. Sixteen applicants were recruited, one withdrew prior to training due to ill-health, and 15 attended the two-day training programme (two males, 13 females), making the recruitment rate 71%. Each training event was attended by between two and nine applicants (mean = 5). Five potential mentors withdrew following training due to illness, unsuitability or other personal reasons (attrition rate 33%).

The resulting ten trained mentors (one male, nine female) actively engaged with the study for between four and ten months. This group became the target sample for the qualitative study at the end of the intervention.

### Qualitative study interview participants

Nine (8 female, 1 male) of the ten active peer mentors agreed to take part in an interview. One mentor had left the study by the end of the intervention. Those who participated were aged between 57 and 75 years (mean= 68, *SD* = 5.4). All had been living with hip/knee OA for at least three years, and five mentors had undergone joint replacement in the year prior to recruitment. All but one had retired, three doing so in the previous two years. Four peer mentors were new to volunteering and five actively volunteered elsewhere. Each mentor supported between one and four mentees, and between them they completed 138 support sessions. Mentor interviews lasted between 48 and 70 min (mean= 60.1).

### Themes

Four overarching themes emerged from the interview data:

- Recruitment;
- Training;
- Mentor-mentee matching;
- Mentorship support sessions.

These themes underpin how peer mentoring support for people with osteoarthritis impacts on their self-management. The results are structured around the four themes, with data from across these themes illustrating mentors’ perceptions of intervention impact on mentees’ OA self-management. Results follow the chronological process of becoming a peer mentor, followed by the practice of being a peer mentor. Pseudonyms along with joint replacement status have been added to the illustrative quotes.

### Becoming a volunteer peer mentor

#### Recruitment

Volunteers’ self-reported motivations for participating in the study included previous volunteering experience, and recently undergoing a joint replacement operation. Volunteers were appreciative of the hospital treatment they had received and felt equipped to help others by sharing information and experiences.

I have quite a… social responsibility in terms of I have this condition, I have benefitted from my hip replacement and therefore… I have some knowledge that I can share. (PM Alison, Joint replacement < 2 years prior to study)

The short-term nature of this role appealed to mentors, as it enabled a low-commitment venture into volunteering. Wanting to “*give something back*” and improve support services for newly diagnosed people were also significant motivators.

I do quite a lot of travelling so I wanted to do something that was quite time limited. (PM Amber, No joint replacement)

One mentor was disillusioned by the lack of support following her diagnosis and wanted to redress the balance; another recognised the possibility of mentoring support as a preventative measure. Most peer mentors considered the role to be potentially interesting and personally informative.

Because I have OA in my knees and hips, I thought I might learn something to my advantage, quite selfishly. (PM Sylvia, No joint replacement)

I thought it might be useful because I’d been through it… I might be able to empathise, and it gave me a chance to learn from you as well. (PM Barbara, Joint replacement > 2 years prior to study)

In relation to recruitment, mentors highlighted the easy application process, and they valued prompt personal responses to their enquiries. Some felt that waiting for safety clearance post-training created a demotivating delay. This loss of momentum led some people to reconsider their motivation to continue, although no-one leaving the study post-training cited this as their reason for withdrawal.

#### Training

Interview data revealed that mentors enjoyed their training. The format was accessible to volunteers from widely ranging backgrounds and they valued the comprehensive content.

I thought the training course was great, you know, and it was interesting on all sorts of levels… it was really well set out, it was very clear. People were very knowledgeable. (PM Amber, No joint replacement)

It was quite an active training and I learned quite a lot as well about osteoarthritis. I thought I knew it all, I didn’t really. I enjoyed it. (PM Karen, Joint replacement > 2 years prior to study)

Mentors used the resource manual and accompanying handouts to structure mentoring sessions and valued it for their own learning.

I really liked the file – I just used to re-read some of that just to keep the brain working. (PM Karen, Joint replacement > 2 years prior to study)

I felt that the information was really, really good because you can apply it to coping strategies. I felt it was all very relevant, all of it was relevant. (PM Jenny, <1 year prior to study)

Mentors appreciated trainers’ professionalism and the varied delivery of material. They also enjoyed the social side of training days, particularly learning from others’ experiences. Suggested improvements to training included input from previously trained peer mentors and more specific information on exercise technique and purpose. The latter was suggested by mentors whose mentees struggled to engage with muscle-strengthening exercises.

Reflecting on the extent to which training prepared them to offer mentorship support, volunteers mentioned relevance of training material and acquiring confidence to support others, but also recognised that it was difficult to be prepared for the individual circumstances of one-to-one sessions.

Well initially after the training I felt very well equipped… Of course, when it comes to the actual thing and you set out for your first meeting… probably wondering what you are walking into and whether you are prepared for it. (PM Andrew Joint replacement < 2 years prior to study)

I think nothing really ever prepares you for that first meeting because people are people. (PM Alison, Joint replacement < 2 years prior to study)

Some also reported that despite feeling that training had prepared them for mentoring, this confidence was affected by the delay in being matched with a mentee. Others indicated that they were less assured of their interpersonal skills or less used to one-to-one volunteering. Comments from mentors with more volunteering experience suggested a greater confidence in supporting people at home.

I was really prepared just to take whatever came really. (PM Karen, Joint replacement > 2 years prior to study)

Yes we knew where we were going, what we were doing, what was relevant to being out there. You could go out with confidence knowing that if you’d any problems you could feed it back. (PM Pamela, No joint replacement)

These mentors understood that training and resources equipped them to deliver support sessions, but making an interpersonal connection was dependent on their skills. Others gained confidence by applying their training firstly to themselves and then adapting it to meet mentees’ needs.

It did, it did equip me yeah, no doubt about that, because of the knowledge I gained and the fact that it made me think about myself and be able to apply it. (PM Jenny, Joint replacement < 1 year prior to study)

Mentor expectations of potential mentees’ needs were guided by training scenarios, although it was emphasised that study participants were self-selecting and had been randomised into the intervention group, meaning mentee support could not be precisely predetermined.

You don’t know what that person’s needs are until you go, do you really. (PM Andrew, Joint replacement < 2 years prior to study)

Preparation for support sessions required a combination of training (expert knowledge, guidance, confidence and skill development) and crucially, peer mentor engagement (interpersonal skills, good communication, adaptability and a person-centred approach).

### Peer mentors’ perceptions on mentor-mentee matching

Twenty-four mentor-mentee dyads were matched; two-thirds were same-sex (two all-male, fourteen all-female) and one-third were mixed. Of the 24 matches, only one mentee requested a different mentor and had to be re-matched.

Mentors’ matching preferences were adhered to as far as practicable. Matching suitability was prioritised but was constrained by mentor availability, and the teams’ limited knowledge of intervention participants. One mentor expressed this succinctly:

Well it’s a difficult one that isn’t it really because if you are doing matching you can only match on what you know can’t you, and that’s a limited pool of knowledge at that point in time. (PM Alison, Joint replacement < 2 years prior to study)

Early in the study the number of mentors exceeded the number of participants recruited to the intervention group. This created an inevitable time-lag between training and matching. However, mentors observed that a delay in getting started was preferable to a poor match and recognised that the “success” of the intervention relied on being well matched.

Definitely yes I think that was the success of it, nothing to do with me or the person… if you are mismatched, nothing is going to work because you are struggling and eight weeks if somebody is struggling is not pleasant, it’s just a chore. (PM Sylvia, No joint replacement)

This mentor measured “success” in terms of relationship experience as well as their perceived ability to offer effective self-management support. Others who were assigned mentees with fewer support needs reported frustration that their time and support was seemingly superfluous.

I did feel as though I wasn’t sure I wanted to do another one, I have to say, after that first one. It’s a lot of effort and… if we’re not getting anything out of it. (PM Barbara, Joint replacement > 2 years prior to study)

Being unable to match dyads in the same location impacted on travel time, which some mentors considered problematic. Although this additional time spent volunteering was perceived as inconvenient, it was generally mitigated by being matched with someone whose company mentors enjoyed and felt they could help. In general mentors reported feeling well matched. They spoke about finding a “*connection*” and being *“fortunate”* to be matched with *“ideal*” people.

And I felt that I’d been very well matched*…* I found with each one really there was a little connection… that I could empathise with or whatever, not just osteoarthritis, other things. (PM Jenny, Joint replacement < 1 year prior to study)

Mentors enjoyed the experience of meeting new people. They looked forward to their visits, became invested in supporting participants and developed an interest in their progress. When this personal connection was harder to achieve, mentors relished the challenge and reported a ‘professional’ concern about their mentees’ progress.

I had two really contrasting situations but that was good that was a challenge, that was interesting, yeah. (PM Karen, Joint replacement > 2 years prior to study)

Mentors welcomed being accompanied to the introductory visit by the VC as it enabled them to make initial observations before committing to support sessions.

You’re aware that you are in somebody else’s space but it was difficult to break through the ice. [VC] cracked through it anyway to start with and listening I was sort of thinking what kind of person they are. I got a lot out of that. (PM Pamela, No joint replacement)

### Reflections on mentorship support sessions

#### Mentoring session format

Mentoring sessions were delivered at mentees’ homes or private workplace. Home visits were universally accepted as preferable and led to high session “attendance”. The private setting and one-to-one format created a comfortable environment in which to share personal information and develop relationships. Mentors reported revealing pertinent details about their experience of living with OA and suggested this aided open dialogue with participants.

I think the one-to-one is important because you sort of expose yourself quite a bit, both, you know, in the kinds of things you discuss. (PM Andrew, Joint replacement < 2 years prior to study)

They wouldn’t feel as if they could open up as much about how pain affected them and how depressed they got… the deeper things were much easier covered if it was one-to-one. (PM Pamela, No joint replacement)

Despite a clear preference for the one-to-one format, mentors acknowledged advantages of group support. Some found the intensity of one-to-one sessions tiring, others noted that motivation to exercise could be more easily sustained in a group setting. Overall, mentors agreed that the advantages of one-to-one, particularly enabling more individualised support, outweighed disadvantages such as higher cost and resource intensity. The majority proposed additional group sessions to enable continued support for some mentees, and to provide an opportunity for mentors to follow-up with mentees.

#### Delivering mentoring sessions

Mentors considered that the timing and frequency of sessions was appropriate for most mentees. Mentors were advised to judge the number of sessions according to mentees’ needs, and plan endings in collaboration with the mentee. Generally, the number of sessions was mutually agreed, although in two cases sessions finished prematurely, with either the mentor or mentee feeling that there should have been more.

Weekly visits were considered important for relationship building and developing routines. When weekly sessions were not possible, the break created an opportunity for mentees to trial their ability to self-manage and enabled both mentors and mentees to prepare for their sessions to end.

Some endings were challenging for mentors, who were concerned for the welfare of their mentees. They were curious to know how mentees would manage after the intervention and felt the loss of the relationship.

He was focused and he was on a journey, but I’ve not had any feedback about him… whether or not he’s kept it up. (PM Karen, Joint replacement > 2 years prior to study)

Occasionally mentees unexpectedly cancelled their last session, leaving mentors feeling unable to complete their planned support.

#### Mentoring session content

Mentors used the educational resource manual and handouts to help structure support sessions with mentees. Interview data revealed that mentees were keen to learn about osteoarthritis, including practical aspects of living with the condition. Mentors were able to draw on their personal experience to encourage preventative measures such as muscle-strengthening exercise, pacing and weight management where continued mobility, pain or joint replacement surgery were of concern.

I think one of the things I got across was maintaining your mobility… that for me I think was really quite fundamental. (PM Alison, Joint replacement < 2 years prior to study)

Mentors commented that some mentees were highly motivated to exercise because they feared losing social connections due to OA-restrictions. Other mentees who were less able to see a direct advantage found muscle-strengthening a chore.

For various reasons they wanted exercise. They wanted to know about it and try to incorporate it better in their daily lives, for different reasons. (PM Jenny, Joint replacement, <1 year prior to study)

She had a lot of difficulty accepting her condition and the potential constraints that it might leave her with. (PM Alison, Joint replacement < 2 years prior to study)

Mentors encouraged mentees to embed muscle-strengthening into daily routines, using regular activity such as waiting for the kettle to boil as a time prompt. Mentors also understood the value of exercise and worked to demonstrate good technique.

Both [mentees] were aware of the strengthening exercises but neither of them were doing them properly. (PM Andrew, Joint replacement <2 years prior to study)

### Goal-setting for osteoarthritis self-management

Goal-setting is a core component of self-management which mentors reported covering regularly with all mentees. However, mentors’ understanding of goal-setting in relation to self-management was variable, and their use of goal-setting as a technique appeared inconsistent. Some mentors struggled to identify and guide mentees towards specific, attainable goals, particularly when they considered goals to be overly ambitious, or faced resistance amongst mentees.

And I think her goal was just to, you know, get over it and not sit and dwell a lot on it. (PM Pamela, No joint replacement)

I was trying to get her to set goals… I was on a hiding to nothing, that was so difficult. (PM Alison, Joint replacement < 2 years prior to study)

Some mentors were unable to accept the importance of goal-setting despite this being an integral part of their training and demonstrated to be effective for OA self-management. These mentors appeared to misunderstand their role as a facilitator, hence determining goals to be unnecessary or mentor directed.

That was kind of tricky in both instances actually because I don’t think you necessarily have to get them to set a goal. (PM Mary, No joint replacement)

Those mentors who adopted a person-centred approach to goal-setting more effectively helped their mentees identify goals and more successfully guided them towards goal attainment.

So I think being a good listener and then just, as I said, following their lead… because I emphasised with all of them that this was for them, this was not about me. (PM Jenny, Joint replacement < 1 year prior to study)

The goals appeared from the people, if you know what I mean. I listened a lot and then [worked] with their specific goals and they changed all the time, that’s okay as well. (PM Karen, Joint replacement > 2 years prior to study)

These mentors understood the importance of listening to what mentees wanted and meeting them at their point of need. Working together mentors and mentees were able to identify challenges and adopt a flexible approach to goal attainment. The dyads who failed to identify specific goals or were too solution focused appeared to have less success.

### Perceived impact of mentorship support for mentees

Interview data revealed that mentors developed a sense of the social, emotional and physical impact of their support on mentees. They spoke about mentees regaining confidence to engage in activity with others outside of the home; to better anticipate their post-surgery capabilities; to share newly acquired OA knowledge with others. Table 2 illustrates the perceived impacts to mentees from mentorship support.

**Table 2.** Perceived impact of mentorship support for mentees.

Sub-theme	Finding	Illustrative quote
Knowledge development around osteoarthritis	Mentees gained better understanding about osteoarthritis as a condition	*When I first went she didn’t know the difference between rheumatoid and osteo and she didn’t know what she had.*
	Mentees gained benefits from osteoarthritis related learning	*His diet really was appalling when I think about it…so it was quite factual information with him about osteoarthritis.*
		*Pacing was a really good one for her and so was pain management.*
	Transfer of knowledge between peers	*My perception was that she got a lot of benefit from my experience of living with the condition and the impact it had on me.*
Acquiring self-management skills	Understanding the significance of muscle-strengthening exercise for daily life	*For her the exercise was very important…because she’s still working…some of her worries were how long can I keep doing this [work] with this condition of osteoarthritis.*
	Mentees developed new skills	*So we brought in effective communication. We talked it through and then she said she felt confident enough to approach her GP with this.*
		*One of the main benefits you get is from doing [exercises] slowly and they hadn’t appreciated that before.*
	Preparing for the future	*I did feel by the end of it that he had made some positive changes and certainly got some stuff out of it for himself going forward should he go ahead and have the surgery.*
Goal attainment	Mentees were helped to identify and set goals	*Neither had sort of realised that they had an issue with balance, but it became clear that they did and that was something we could work on.*
		*It did become a goal to be able to manage the stairs better, yes definitely.*
	Mentees embedded exercise into their daily life	*[She] once said to me I can do these [exercises] holding on to the work surface waiting for a pan of potatoes to boil.*
	Mentees achieved their goals	*She had set a longer-term goal about going swimming. Well she had actually gone back to swimming by the time we finished sessions so that for me was really… I felt like a proud mother.*
		*Three weeks later when I said to her how’s the knee, have you noticed any improvement with the exercise? She actually went ‘Yeah, look at this.’ She could lift it!*
Psychosocial benefits	Mentees and mentors developed trusting relationships	*At least he could be more vulnerable with me as well, he could say when he’d been up all night with his knees.*
	Mentees developed confidence	*And this is the lady that eventually at the end said I’m going to take these exercises to the older ladies at the church….because a lot of them have got osteoarthritis.*

Additionally, mentors noted that mentorship support allowed mentees to voice their concerns about the overriding impact of OA on their lives. Mentors judged that addressing concerns about OA was particularly important for mentees who had difficulty accepting their condition and reflected that in some cases the impact of their support was more subtle, but no less powerful, than goal attainment.

A key motivator for peer mentors was perceiving that their support impacted positively on their mentees. Mentors displayed satisfaction in the role when they were able to identify clear self-management improvements.

The reason for doing it… well, from my point of view, was to make people handle it better, not be frightened and to have the confidence… and now I think she feels more confident to actually take out what we’ve shown her, especially the exercises, to other people, which is fantastic. (PM Pamela, No joint replacement)

## Discussion

This qualitative evaluation is novel in that it reports on the combined process of engaging and training volunteers to become peer mentors; and the feasibility, acceptability and value of being a peer mentor to support others’ self-management of OA.

This study found that:

- It is both feasible and acceptable to engage older volunteers with OA as peer mentors;
- Mentors perceived their involvement to be personally beneficial; and
- Mentors judged their mentorship support had a positive impact on the self-management of their mentees.

### Elements of a successful intervention

The success (experience and health outcomes) of a mentorship support intervention depends on its components [25], style of delivery and how far it addresses the needs of the target population [23]. Sustainability depends on personal attributes of its actors, and the reciprocity of their relationships [45]. Fundamental to the continued engagement of our peer mentors was them finding the intervention acceptable and that their contribution had value. Their contribution as “peers” meant that they understood the impact of OA on daily life and significance of sustainable behaviour change for ongoing self-management. Mentors who optimised opportunities to share their experiences and motivate mentees, for example, to embed exercise and activity in daily routines, perceived the greatest impact on mentees’ self-management.

Matching of mentors with mentees is an integral element of successful mentorship support, yet it has been given little attention in the literature. Commonly, studies have reported on matching criteria such as gender, age, and symptom presentation but have not always evaluated the usefulness of matching on these criteria. However, other studies suggest that matching peers based on common interests and personal attributes, more positively contributes to the success of an intervention [29,30,36]. A short-lived intervention, such as that reported here, requires efficient development of trusting relationships, rapport and reciprocity between mentors and mentees. In our study, taking account of personality traits during matching helped facilitate relationship development which in turn impacted on mentee accountability.

### Recruitment of volunteer peer mentors: strengths and limitations

This study established that it is feasible to recruit community-based volunteers. Recruitment and attrition rates for peer mentors were comparable to other studies [25,35] and those who became active mentors were well motivated and invested in making a positive difference to their mentees. One limitation of the recruitment strategy was that it resulted in relatively few enquiries despite its wide potential reach. Better use of social media and community publications would have been valuable.

The “peer” element required that we recruit “older volunteers” (aged 50+), most of whom were retired and seeking to use their skills and life experience to help others. Given an abundance of other volunteering opportunities for this cohort, it was important to convert tentative enquiries into applications. This was helped by having an easy and responsive application process and may have been improved by offering more flexibility with training dates.

Both new and experienced volunteers were attracted to the role and overall reported having a rewarding experience. They enjoyed meeting new people (mentees and other mentors) and felt well supported by the study team and fellow volunteers. Retaining our trained volunteers created a significant advantage as evidenced by the confidence and effectiveness with which they supported mentees.

One challenge of recruiting people with OA as peer mentors is that they are facing their own self-management issues, potentially affecting attrition and the reliability with which they can provide mentorship support. In our study, withdrawal was highest immediately following training possibly because these sessions highlighted key role requirements and expectations of peer mentors. Some may have reflected on their ability to meet the demands of the role. Incorporating a more stringent application process may have helped both the study team and volunteers assess their suitability for peer mentoring prior to training.

### Peer mentor training: strengths and limitations

Mentorship training in health self-management varies widely in duration, format, and scope. Key elements of quality training include condition-specific knowledge and self-management techniques; mentoring safety and skills; and progression tracking through goal-setting. Training efficacy is enhanced by enjoyment and opportunities for socialisation [9,25,26,28]. All of these elements were incorporated into our training programme. Mentors rated training highly, appreciated the specialist input, and the comprehensive coverage of topics which varied in presentation. They valued the structure and content of the resource manual both in training and for use in sessions.

Through training, mentors gained new knowledge about OA and learned self-management tools and techniques which they applied to themselves. They personally benefitted from this, and in testing out new knowledge and techniques mentors acquired confidence and legitimacy for mentorship support sessions.

Goal-setting is an important self-management tool [46,47], yet the significance of using this technique for effective self-management was not adequately understood by all mentors. Problems included focusing on non-specific goals; setting goals for mentees rather than enabling them to identify their own goals; and rejecting setting goals as being overly didactic. This area of training could be improved by emphasising the value of goal-setting for effective self-management, and by enhancing mentors’ skills to guide mentees towards goal attainment. Our regular mentor group sessions helped maintain engagement and provided opportunities for the study team to offer guidance. Refresher training sessions would have been useful to ensure continued skill development.

### Matching of mentor-mentee dyads

Despite having a limited pool of available mentors, it was possible to match and support all study participants within the intervention period. In a real-world setting, recruitment of mentors and mentees would be ongoing, attrition more easily counteracted and matching more easily regulated. Recruitment of study participants (mentors and mentees) was unintentionally staggered. This created a delay in matching and starting the intervention. One consequence of this was requiring mentors to simultaneously support more than one mentee in order to meet study deadlines. It may have been prudent to set an expectation at the outset that mentors would be matched with at least two mentees.

Although unavoidable, some mentors were discouraged by the delay between training and being matched with a mentee. This was addressed by providing regular study updates and encouraging attendance at mentor group sessions, through which they could learn about others’ experiences of mentoring.

The original matching criteria for this study were adjusted to become more flexible and to incorporate interpersonal characteristics, as well as common interests and life circumstances. Although we adhered to mentor/mentee’s gender preferences in matching, as the study progressed, we recognised that personality was often a more relevant matching characteristic, where, for example, strong characters were more appropriately matched together.

As with other studies [48], development of mutually rewarding relationships inevitably meant that some mentors found endings and associated loss of the relationship difficult. Although they understood the boundaries of the mentor-mentee relationship, this did not account for the emotional transition from weekly interaction to no interaction. Mentors wanted feedback on their mentees’ progress. This was due to concern for the mentee, and a desire to reinforce their value to the intervention. The Volunteer Coordinator offered a telephone debrief to mentors after each closing session, but could have gone further to facilitate the transition by attending part of the final support session, as has been done in other studies [30].

### Mentoring session delivery

Mentors and mentees effectively arranged timing and frequency of sessions between themselves. Holding sessions at the mentee’s home or workplace helped reduce non-attendance, created a comfortable environment and was reported by mentors to be an acceptable format. Mentors readily complied with the buddy system and recognised that it was an essential mechanism for their safety and that of their mentees. Travel burden was a problem for some mentors and session frequency was sometimes disrupted by health issues. To overcome this, the face-to-face format could be interspersed with telephone or online sessions. Although remote support does not allow for the quality of personal connection attained through face-to-face meetings, in recent times many people have adjusted to connecting socially online. Adapting the intervention to include an online option would reduce practical barriers to session delivery and may enhance inclusion of potential mentors restricted by health or mobility, although a move towards online sessions may pose further challenges created by digital exclusion [49].

Mentors enjoyed the autonomy of delivering one-to-one sessions and selecting appropriate session content within the structure of the core and optional topics. This enabled them to provide tailored support to mentees, focusing on goals and motivational techniques in order to flexibly address support needs.

### Perceived value of mentorship support

The central purpose of a mentorship support intervention is that it impacts positively on self-management behaviour of mentees as well as providing tangible benefits to volunteer mentors. Mentors in this study gained enjoyment, increased confidence, knowledge and skills, and wanted to feel that their involvement added real value. Critical to their satisfaction and continued engagement was believing that their support impacted on OA self-management. Mentors’ perceptions of what mentees gained from the intervention indicates that in general they judged their support to be meaningful. As one mentor explained: *“I gained the pleasure and feeling that I was doing some good, which is what volunteering is*”.

Notable exceptions occurred with some mentees who had lower support needs, and for whom the intervention was less appropriate. Mentors in these cases became frustrated that their support was not used to best effect, and together with their mentees, reframed the purpose of their involvement as ‘helping with research’ rather than addressing OA self-management. In this way they gained enjoyment from the role and reported that both they and their mentees benefitted from the interaction and shared experience.

## Conclusion

This study demonstrated that it is feasible to recruit, engage and train community-based volunteers to offer peer mentorship support to people with OA. Recruitment and attrition targets were achieved, and suitably skilled people were attracted to the role, although the recruitment process could be improved by better placed advertising, using multiple sites and offering additional opportunities to join training.

Training was well-received, and adequately equipped volunteers to become OA peer mentors. Ongoing support enhanced acceptability and provided opportunities for development in the role. Better use of goal-setting as a self-management technique could be emphasised.

Mentor-mentee matching was a significant factor for feasibility and acceptability of this intervention. Matching was a more complex process than originally anticipated, relying on perceptions and awareness of the study team and adaptability of mentors. It was further hampered by mentor availability. Plans for a larger study should include consideration of the matching process in relation to recruitment and availability of mentors, as well as the appropriateness of the match.

Feeling supported in the mentoring role and identifying benefits derived through participating in the intervention were essential aspects of acceptability for our peer mentors. Recognising that their support impacted on self-management of mentees was fundamental to the success and sustainability of our intervention.
